# Hydrographic data inspection and disaster monitoring using shipborne radar small range images with electronic navigation chart

**DOI:** 10.7717/peerj-cs.290

**Published:** 2020-09-14

**Authors:** Jin Xu, Baozhu Jia, Xinxiang Pan, Ronghui Li, Liang Cao, Can Cui, Haixia Wang, Bo Li

**Affiliations:** 1 Maritime College, Guangdong Ocean University, Zhanjiang, Guangdong, China; 2 Navigation College, Dalian Martime University, Dalian, Liaoning, China; 3 Marine Engineering College, Dalian Maritime University, Dalian, Liaoning, China; 4 Civil Aviation College, Shenyang Aerospace University, Shenyang, Liaoning, China; 5 Laboratory Department, Liaoning Hydrogeology and Engineering Geology Reconnaissance Institute, Dalian, China

**Keywords:** Shipborne radar, Information fusion, Electronic navigation chart, Oil spill

## Abstract

Shipborne radars cannot only enable navigation and collision avoidance but also play an important role in the fields of hydrographic data inspection and disaster monitoring. In this paper, target extraction methods for oil films, ships and coastlines from original shipborne radar images are proposed. First, the shipborne radar video images are acquired by a signal acquisition card. Second, based on remote sensing image processing technology, the radar images are preprocessed, and the contours of the targets are extracted. Then, the targets identified in the radar images are integrated into an electronic navigation chart (ENC) by a geographic information system. The experiments show that the proposed target segmentation methods of shipborne radar images are effective. Using the geometric feature information of the targets identified in the shipborne radar images, information matching between radar images and ENC can be realized for hydrographic data inspection and disaster monitoring.

## Introduction

A shipborne radar is mainly used to navigate and avoid obstacles in areas such as port accesses and narrow channels and is becoming an important piece of equipment in modern navigation. It can also provide functions over a long distance, such as ship positioning, shoreline monitoring, and rescue location. In particular, it can function in rainy and foggy weather and other harsh environments continuously (Lisowski, 2019). A shipborne radar is also an effective remote sensing tool that can view the scene in real time and provide auxiliary observations in maritime accidents. In the early 1980s, the International Maritime Organization (IMO) stipulated that radars should be one of the necessary equipment for navigation (Capria et al., 2017). With continuous progress and improvement, shipborne radars have been equipped with the function of automatic radar plotting aids, which can provide the course, speed, distance, and orientation of the target ship. They can also perform the functions of automatic identification system (AIS) target fusion, electronic navigational chart (ENC) overlay display, and network monitoring.

An ENC can identify the type and location of a static target. Canada, the USA, Japan, and other countries have promoted the use of ENCs as the core equipment of ship navigation (Zhou et al., 2005). Countries around the world have successively carried out research on ENCs. The International Hydrographic Organization, International Electrotechnical Commission and other relevant international organizations have steadily released S-57 (Transfer Standard for Digital Hydrographic Data), S-63 (Data Protection Scheme), S-100 (Universal Hydrographic Data Model), and S-102 (Bathymetric Surface Product Specification), among others. At present, an increasing number of companies can produce ENC application products that meet international standards, such as Transas of Russia, Sperry of Norway, Offshore of Canada, Atlas and 7cs of Germany, and Raytheon of the USA.

In the 1850s, some scholars proposed the advantages of data fusion of radars and traditional paper charts; they also proposed methods for manual identification (Home, 1952; Dickson, 1952). The information fusion of shipborne radars and ENCs cannot only effectively compensate for the inability of radars to provide water depth data and judge the type of obstacles but also provide the comprehensive situation of the real static and dynamic targets around the ship, greatly improving navigation safety. For example, Kazimierski and Stateczny put forward a method to fuse shipborne radar and AIS information in Electronic Chart Display and Information System (ECDIS) to avoid collision for navigation safety (Kazimierski & Stateczny, 2015). In addition, the target information monitored by shipborne radars can be displayed in the ENCs of ships and shore-based command centers through data fusion technology and wireless networks, which is convenient for a unified action and collaborative operation (Donderi & Mcfadden, 2003). This information fusion also has the following advantages:

(a) The information fusion of oil spills is conducive to the regular monitoring of law enforcement ships or emergency responses. Network monitoring can effectively assist quick disposal.

(b) In maritime search and rescue, radars also show a strong applicability. It can integrate the target information in distress into an ENC so that the command center can quickly dispatch and carry out rescue operations.

(c) As the sea moves, the coastline changes. It is very important for ship collision avoidance ability to monitor the target information of the continental and island coastlines in real time. However, due to altitude limitations, radars are not fully suitable for detecting the entire island. At this time, it is necessary to overlay the object information identified in the ENC to determine an accurate cruise route.

(d) Because the updating of ENC data is periodic, the fusion of radar data can also verify the data effectiveness of the ENC and hydrographic production.

With the release of the S-57 standard by the IMO in 1995, ENCs have played an important role on the ships (Weeks, 1993; Alexandrov, Draganov & Kolev, 1995). Companies in Germany, the UK, Canada, Russia, and China have attempted to achieve the superposition of ENC and radar images (Kolev, Alexandrov & Draganov, 1995; Tang & Shi, 2008; Yang, Dou & Zheng, 2010; Chang, Lai & Hsu, 2014; Wang & Huang, 2014). For example, in 1994, the Offshore ECDIS-3000 system of Canada realized the image superposition of ENCs and radars to ensure navigation safety in foggy weather. Because of many factors, such as low technology level, poor hardware facilities, the huge amount of information in ENCs, and heavy processing of radar signals, the efficiency of data fusion was low (Cai, 2011). Later, Hamilton (Norwegian) and Transas made continuous breakthroughs to improve the efficiency of data fusion (Liang, 2010). At present, some products can realize the rapid information integration of shipborne radars and ENCs, such as the dKart navigator from the USA (Cao, 2017). However, due to commercial competition, their technology has not been open to public. Transas and Sperry have developed a mature radar signal processing card, which can be directly installed in the PCI slot of a general PC. When a radar signal processing card is connected to the radar’s receiving and transmitting antenna unit, it can directly process the radar video signal, record and track the moving target, and control the transceiver (Wang, 2017). In this way, the radar video signal can be transformed into an image and integrated into an ENC through secondary development.

The total shipborne radar data were mapped to the ENC at the outset, so the processing efficiency was very poor. In the 2000s, many scholars found that only the target identified by the shipborne radar should be integrated into the ENC to improve the efficiency of data processing (Lin et al., 2009; Oh, Kim & Jeong, 2015). We divided radar targets into bright and dark in this paper. The strong reflectors of radar electromagnetic waves are called bright targets, such as ships, islands, and shorelines. Bright targets are often used for navigation and collision avoidance. Nishizaki et al. (2014) tried to automatically segment ship targets through image processing and a cluster analysis using shipborne radar images. The automatic detection accuracy in images with ships was high. However, false positive targets were always detected in images without ships. Hu (2018) divided shipborne images into image blocks after preprocessing. He used a deep learning recognizer to classify radar image blocks and realize the classification of ships, shorelines, sea clutters and backgrounds. This method can achieve a better recognition effect. However, ships and land are bright targets, and wrong classification results were obtained when both existed in the image. Wang et al. (2020) applied an automatic multilevel threshold method to extract bright targets in a shipborne radar image with fine preprocessing. The experimental results of this method are excellent. However, the efficiency is relatively low because of the time required to compute a multilevel threshold. The reflectors that can weaken the radar electromagnetic echo are called dark targets, such as oil spills. Dark targets are mostly used for monitoring marine disasters. Zhu et al. (2015) proposed a manual single-threshold method to extract oil spills after adjusting the whole gray level of the shipborne radar image. This method can adjust the gray distribution of the sea clutter area, and it has a certain reference significance for dark target extraction. Based on their study, Xu et al. (2019) proposed the application of a contrast enhancement algorithm and local adaptive threshold to extract oil spills, making dark targets easier to identify.

In this paper, we propose a dual-threshold method to segment bright targets and the local adaptive threshold to extract dark targets in shipborne radar images. Then, the targets are mapped into the ENC, providing technical support for hydrographic data inspection and disaster monitoring.

## Materials & Preprocessing Methods

### Materials

**Figure 1:**
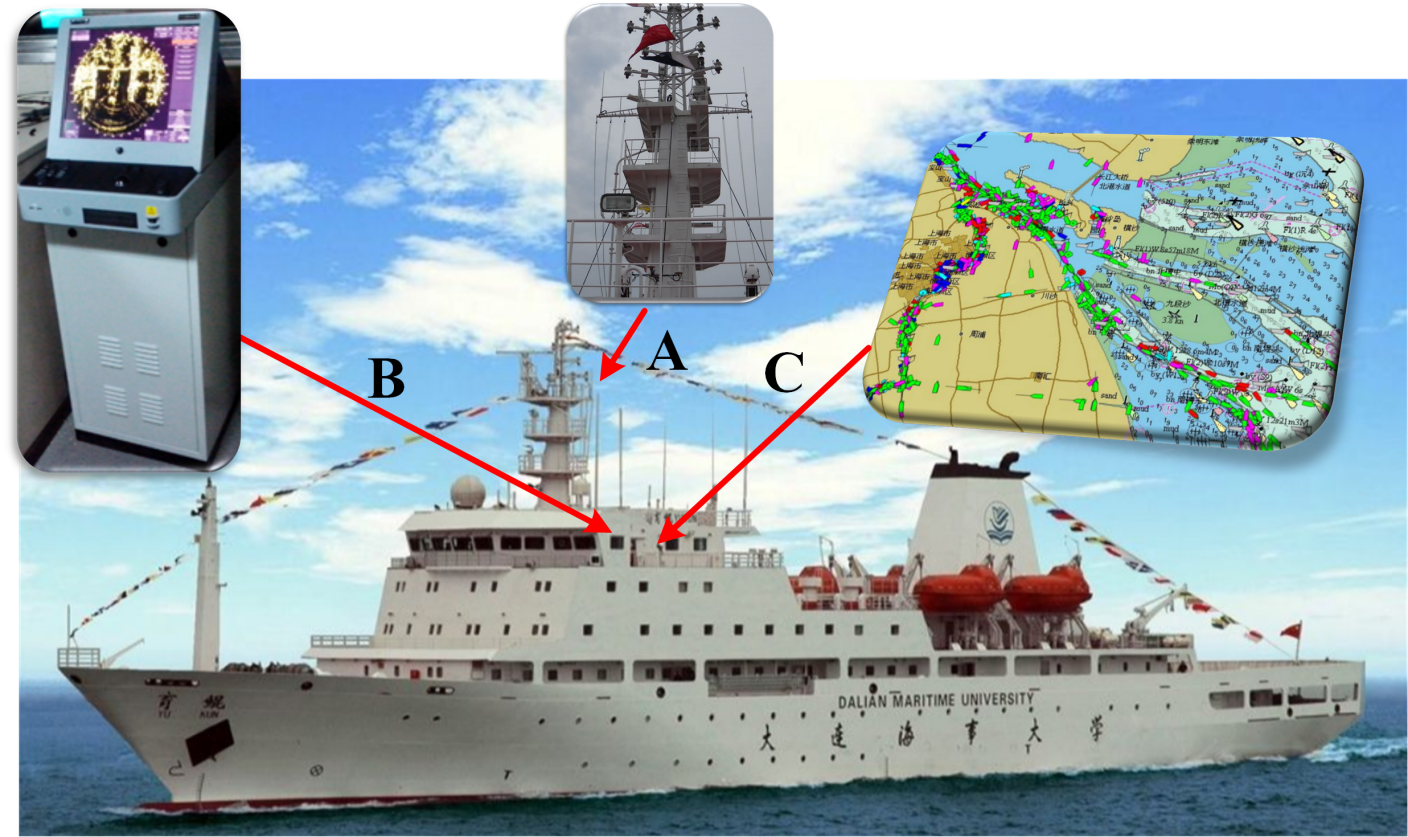
Hardware architecture. (A) Installation position of radar antenna. (B) Radar Image Acquisition System. (C) ENC.

**Figure 2:**
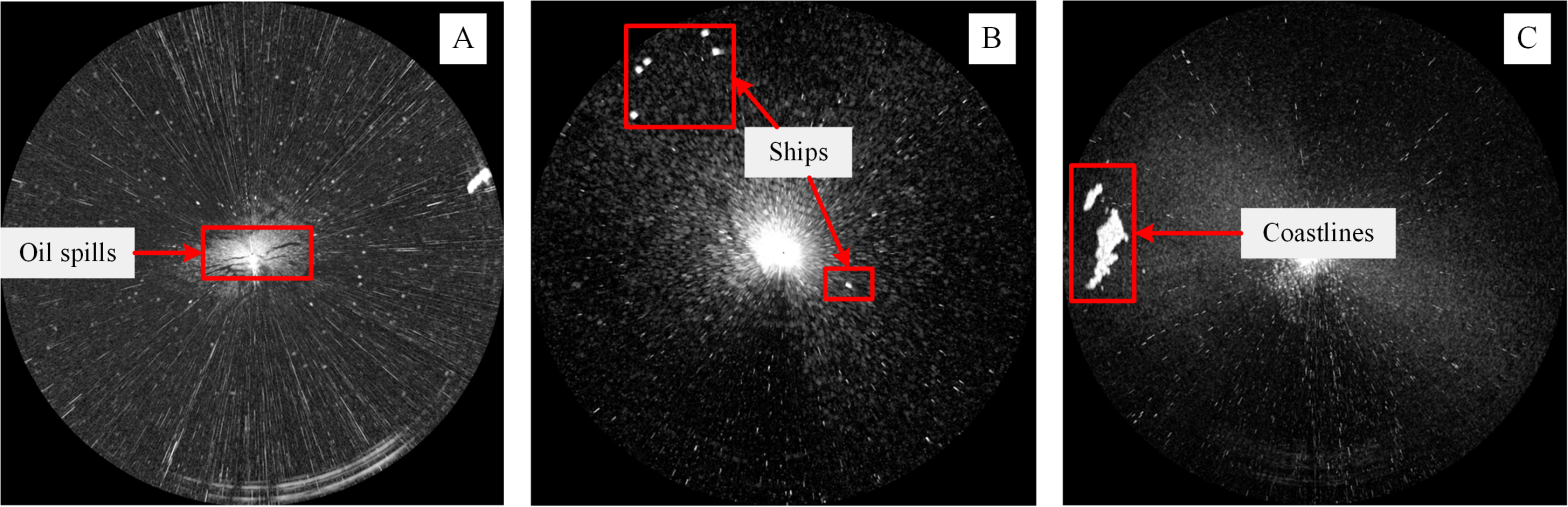
The original radar images. (A–C) are acquired at 23:20:27 21th July, 2010, 17:27:44 10th August, 2015, and 08:59:20 12th August 2015, respectively.

**Figure 3:**
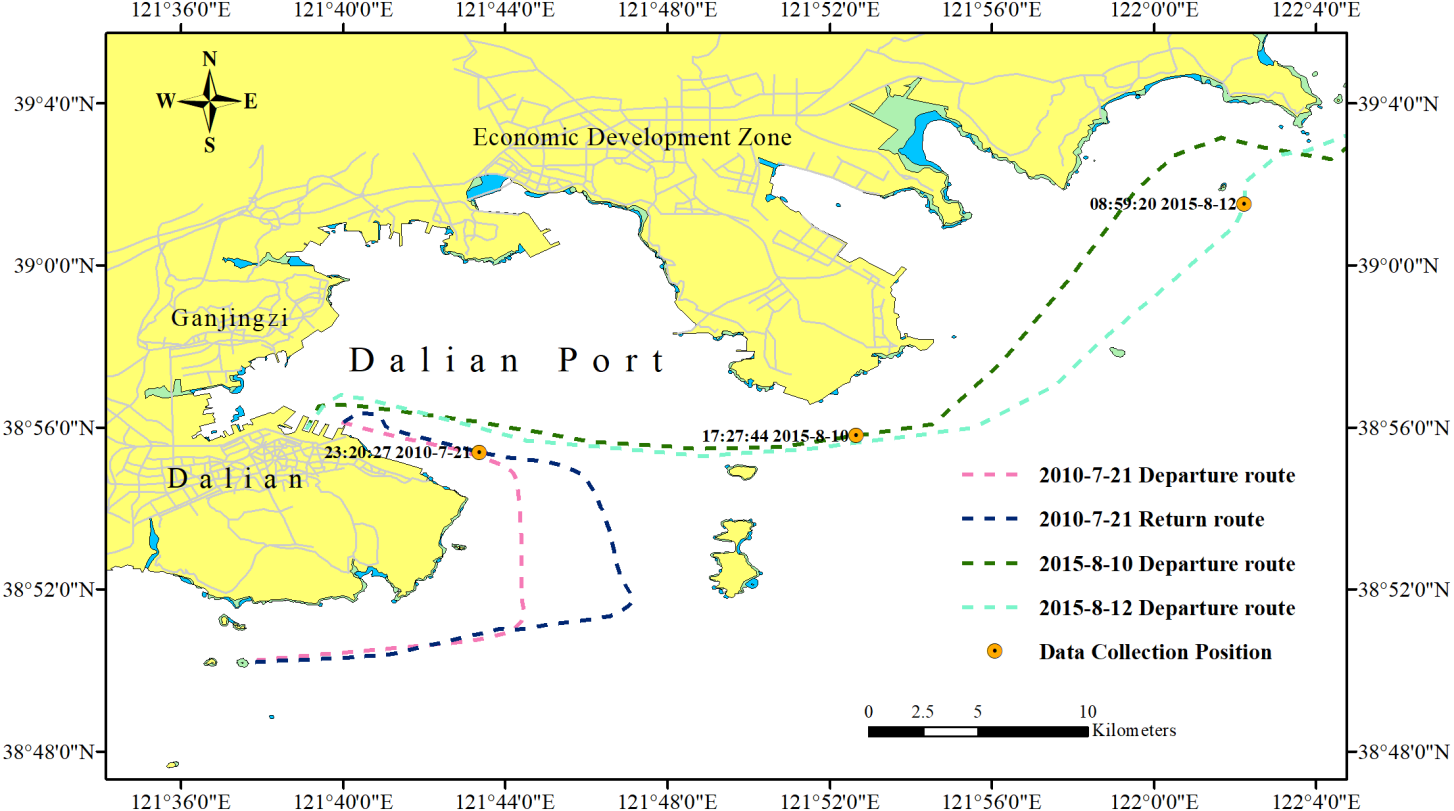
Chart of Dalian Port.

32 shipborne radar images were acquired during the cruises of the teaching-training ship *Yukun* (Fig. 1) of Dalian Maritime University. Among them, 21 images contained ships, 7 images contained shorelines, and 6 images contained oil spills. The three images shown as Fig. 2 are used to introduce our method. The radar parameters are listed in Table 1. The data acquisition period is 2 s. The monitoring radius in the images is 0.75 nautical miles (NM). The image size was 1,024 × 1,024 pixels and the gray level was 256. The chart of the Port of Dalian is used here, as shown in Fig. 3. The image processing and analysis platform is MATLAB 2014a, and the system development platforms are Visual Studio 2010 and ArcGIS 10.2.

### Data preprocessing

**Figure 4:**
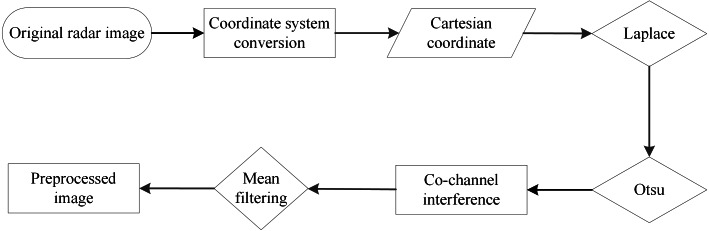
Flow chart of data preprocessing.

Many oil films exist around the ship, as shown in Fig. 2A. Ships and coastlines information are presented in Figs. 2B and 2C. The images needed to be denoised first, especially the co-frequency interference noises, which seriously affect the uniform gray distribution. The data preprocessing scheme is shown in Fig. 4. First, the image was converted to a Cartesian coordinate system. After that, the co-frequency interference noises were segmented by the Laplace operator and Otsu method. They were then suppressed via mean filtering.

**Figure 5:**
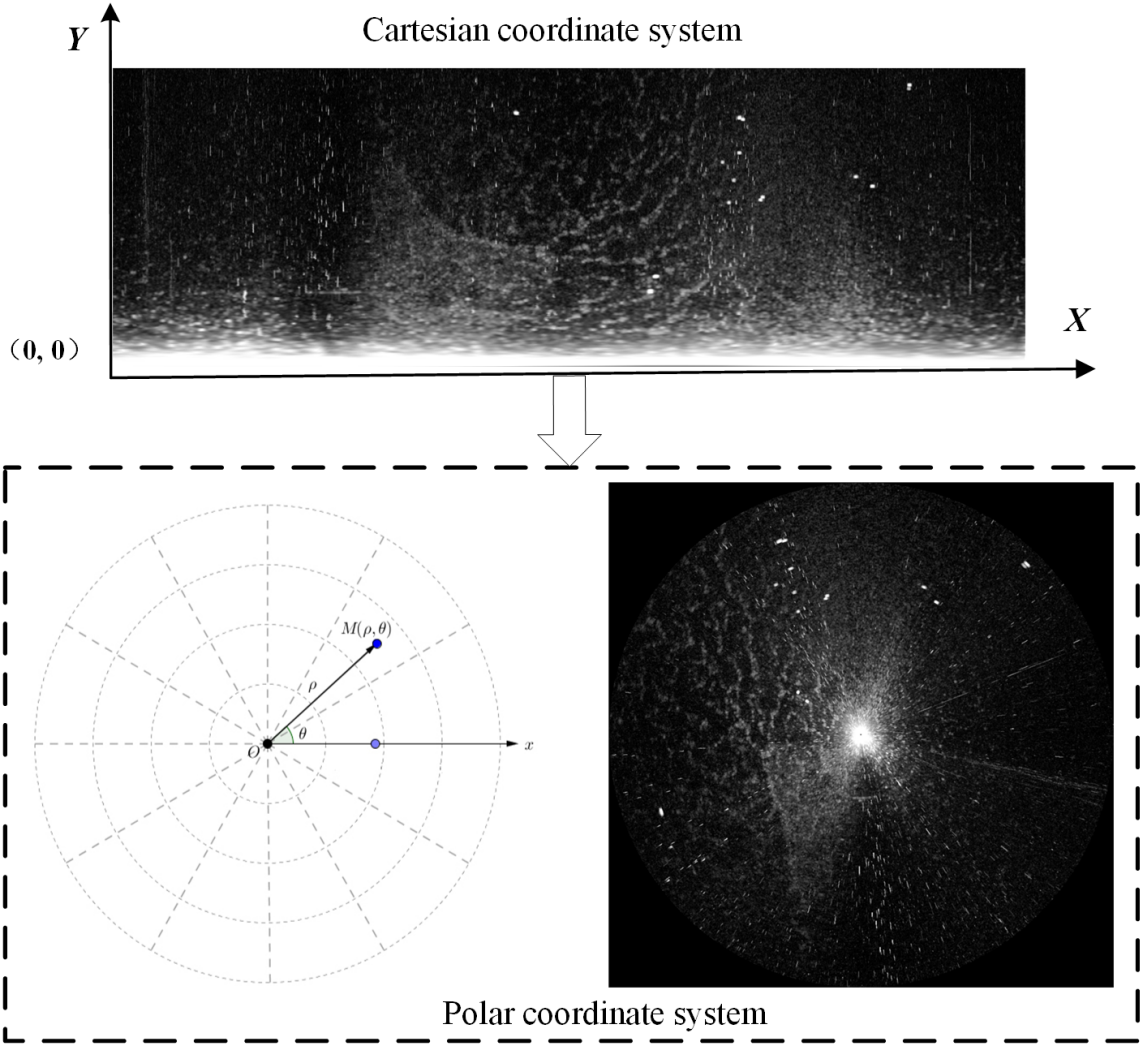
Transformation of coordinate system.

The original shipborne radar image was collected in the Cartesian coordinate system. In Formula Eq. (1), the image is transformed into a polar coordinate system for understandable, as shown in Fig. 5.

(1) \begin{eqnarray*} \left\{ \begin{array}{@{}l@{}} \displaystyle \rho =\sqrt{{x}^{2}+{y}^{2}}\\ \displaystyle \theta =\arctan \nolimits \left( \frac{y}{x} \right) \end{array} \right. ,\end{eqnarray*}

where *x* and *y* are the abscissa and ordinate of the Cartesian coordinate system, respectively, and *ρ*, *θ* are the distance and orientation of Polar coordinate system, respectively.

**Figure 6:**
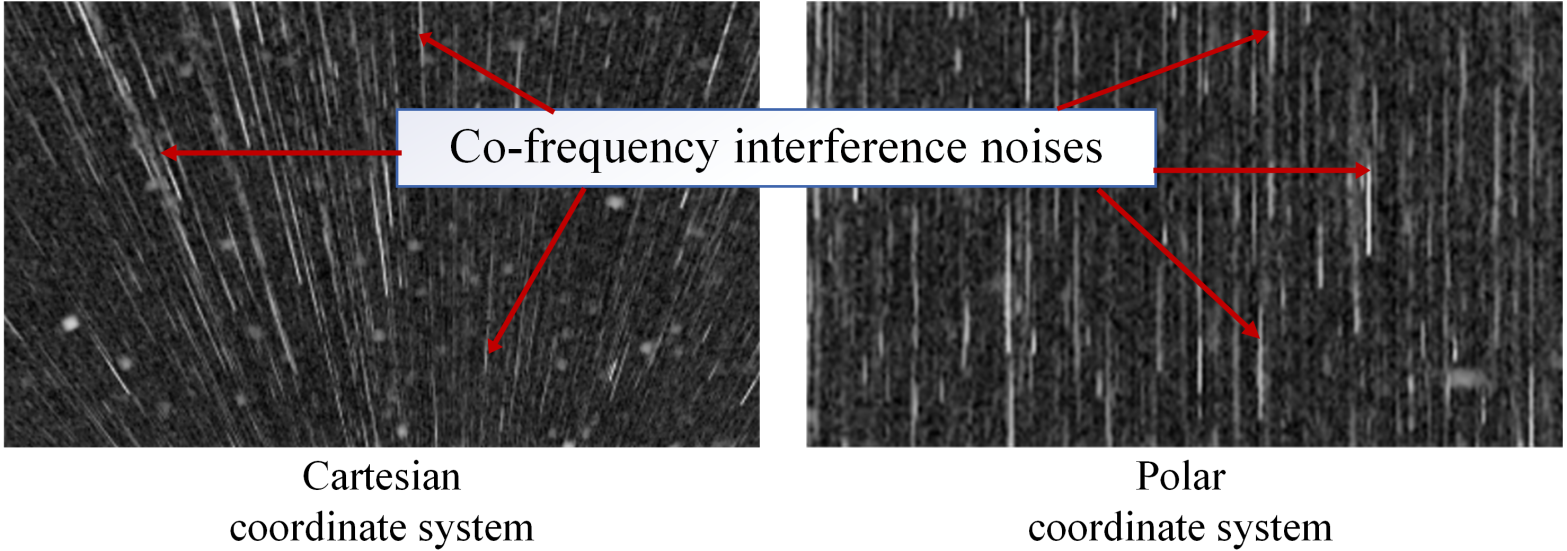
Co-frequency interferences in two coordinate systems.

Many meaningful image features are present in the Cartesian coordinate system. For example, the co-frequency interferences appear as radial noises in the vertical direction (Fig. 6), and they are processed without considering the angle factor. Therefore, we used Formula Eq. (2) to transform the experimental data from the polar coordinate system to the Cartesian coordinate system.

(2) \begin{eqnarray*} \left\{ \begin{array}{@{}l@{}} \displaystyle x=\rho \cos \nolimits \theta \\ \displaystyle y=\rho \sin \nolimits \theta \end{array} \right. ,\end{eqnarray*}

The Laplace operator is as follows:

(3) \begin{eqnarray*}{\nabla }^{2}f=4f(x,y)-[f \left( x+1,j \right) +f \left( x-1,j \right) +f(x,\mathrm{y}+1)+f \left( x,y-1 \right) ],\end{eqnarray*}

where *x* and *y* are the abscissa and ordinate of the image, respectively. The mean filtering is calculated (*n* = 3 in our experiment) as

(4) \begin{eqnarray*}f(n,y)= \frac{\sum _{i=0}^{n}X(n-1,y)+X(n+1,y)}{2n} ,\end{eqnarray*}

**Figure 7:**
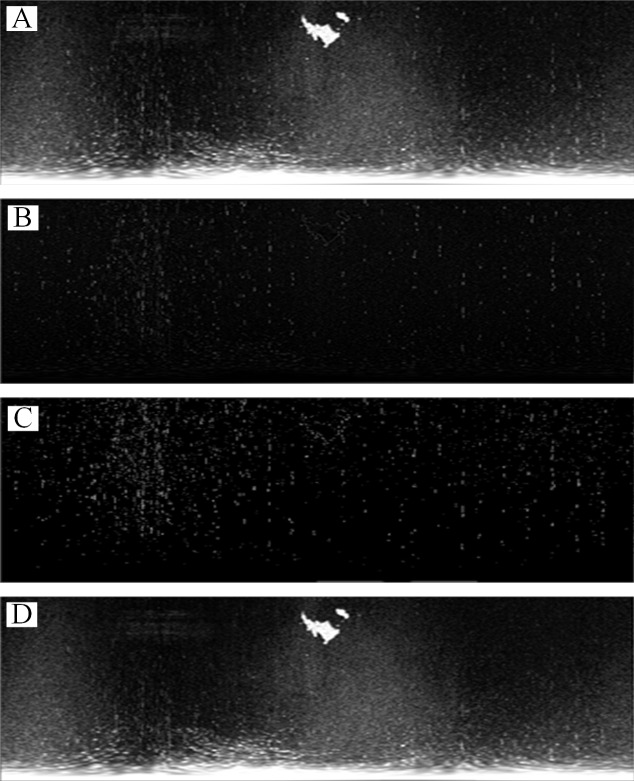
The preprocessing method. (A) Coordinate system transformation. (B) Laplace operator convolution. (C) Otsu automatic threshold segmentation. (D) Mean filter.

Figure 2C is used to introduce the preprocessing flow, as shown in Fig. 7. First, the original radar image was transformed from the polar coordinate system to the Cartesian coordinate system (Fig. 7A). Then, the Laplace operator was used to enhance the co-frequency interference and weaken the other information (Fig. 7B). Next, the Otsu threshold was used to segment the co-frequency interference (Fig. 7C). Finally, a mean filter was used to suppress the co-frequency interferences, as shown in Fig. 7D.

**Table 1:** Parameters of shipborne radar.

Parameter	Value
Product type	Sperry Marine B.V.
Band	X-band
Detection distance	0.5∕0.75∕1.5.3.6.12 NM
Image size	1,024 ×1,024
Antenna type	Waveguide split antenna
Polarization mode	Horizontal
Horizontal detection angle	360°
Rotation speed	28–45 revolutions/min
Length of antenna	8 ft
Pulse repetition frequency	3000 Hz/1800 Hz/785 Hz
Pulse width	50 ns/250 ns/750 ns

## Results

### Data analysis process

**Figure 8:**
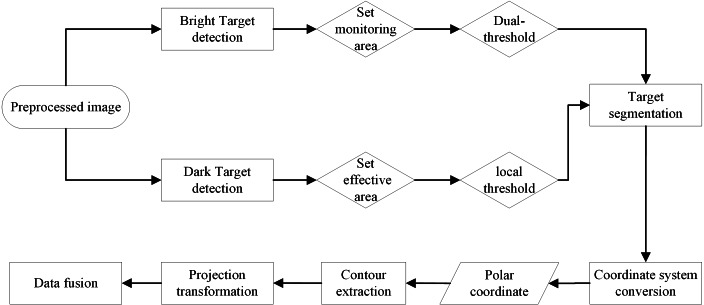
Data analysis process.

The data analysis process is shown in Fig. 8. First, it is necessary to determine whether to extract the light or dark target firstly. For a bright target detection, the dual-threshold method is used for segmentation after setting the monitoring area. Otherwise, the local threshold method is used to segment the dark target after setting the effective wave area. Then, the identified targets are transformed to the polar coordinate system. After that, the contour of the target is extracted, and the boundary points of the contour are projected onto the geographic coordinate system for data fusion.

### Bright target segmentation

**Figure 9:**
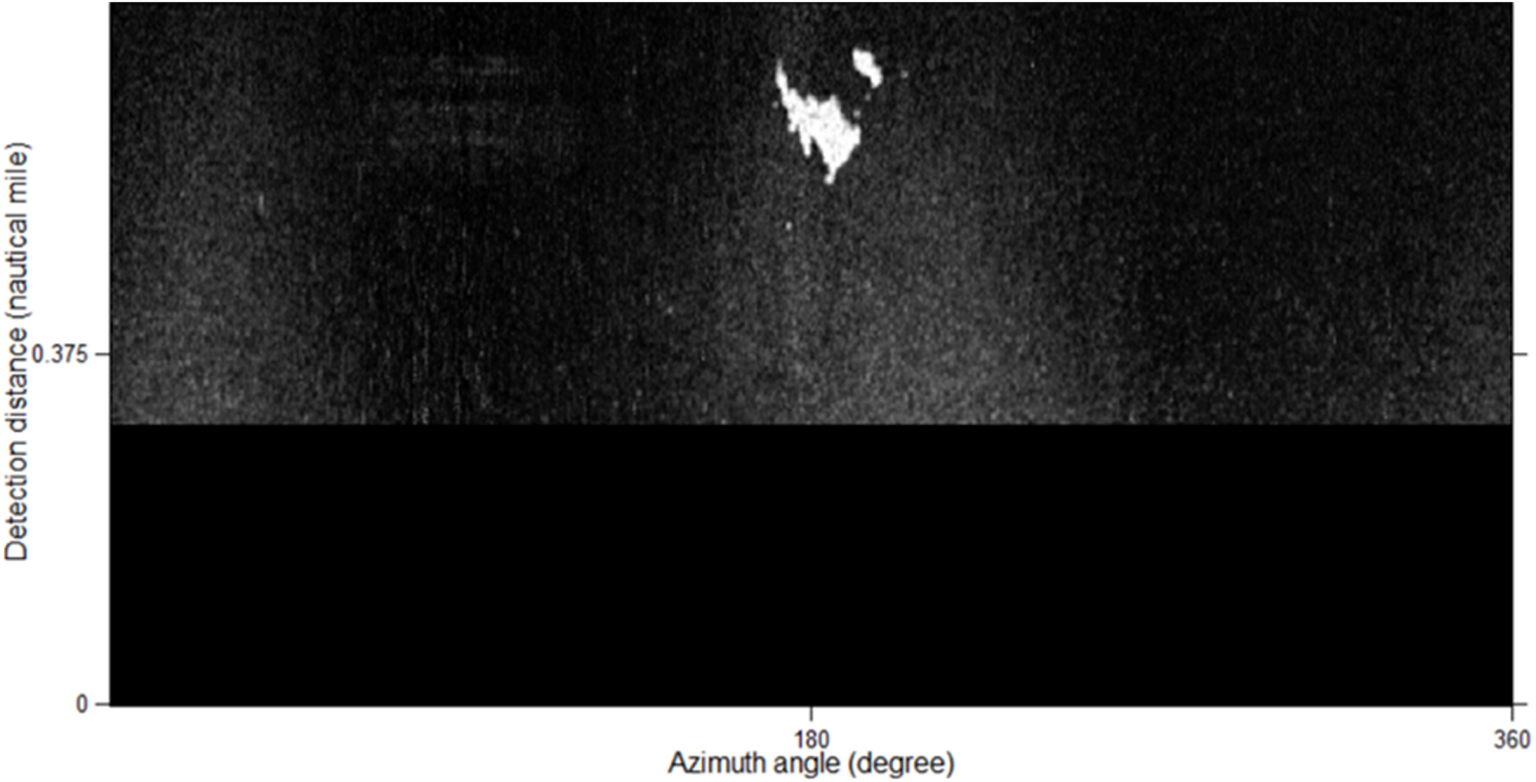
Monitoring area is set between 0.3–0.75 NM.

In the original shipborne radar image, the brightness of the wave echo area at a distance is high. Therefore, the monitoring area should be set before identifying the ship or coastline. We removed the image information within 0.3 NM (mainly the highlighted wave echo pixels) and retained the image information of the bright target monitoring area, as shown in Fig. 9.

**Figure 10:**
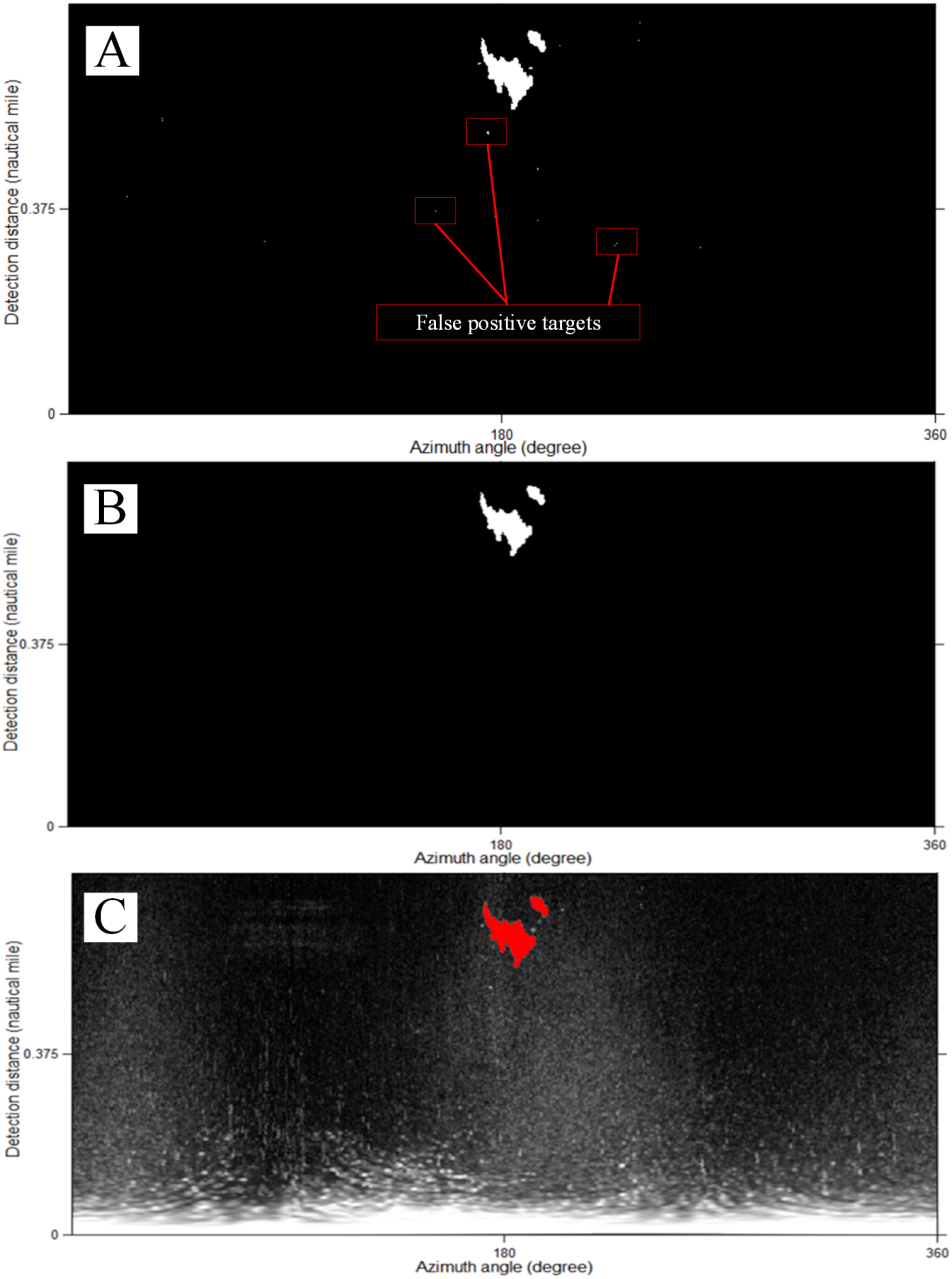
The dual-threshold segmentation. (A) Gray threshold segmentation of *T* (*gray*) = 120. (B) Pixel number threshold segmentation of *T(Area)* = 20. (C) Composite image.

The following dual-threshold method is selected to extract the target, as shown in Fig. 10.

(5) \begin{eqnarray*}D(x)= \left\{ \begin{array}{@{}l@{}} \displaystyle d(x)\gt T(gray)\\ \displaystyle A(region)\gt T(Area) \end{array} \right. ,\end{eqnarray*}

where *D* (*x*) is the continuous pixel of the target, *d* (*x*) is the gray value of the image pixels, *T* (*gray*) is the gray threshold, *A(region)* is the number of pixels of the continuously highlighted image regions, and *T(Area)* is the number threshold of *A(x)*. For a radar image of 1,024 pixels ×1,024 pixels with a monitoring range of 0.75 NM, the actual area of 1 pixel is 7.34 m^2^. It is suggested to set the continuous highlight area of less than 20 pixels as speckles and eliminate them here. If the radar range increases and the image size does not change, then *T(Area)* should be smaller.

### Dark target segmentation

Because of the different reflection characteristics of the monitoring targets, the identification of oil spills is more complicated than that of ships and coastlines. The oil film is dark in the sea clutter area, so the effective monitoring of the wave should be determined first.

**Figure 11:**
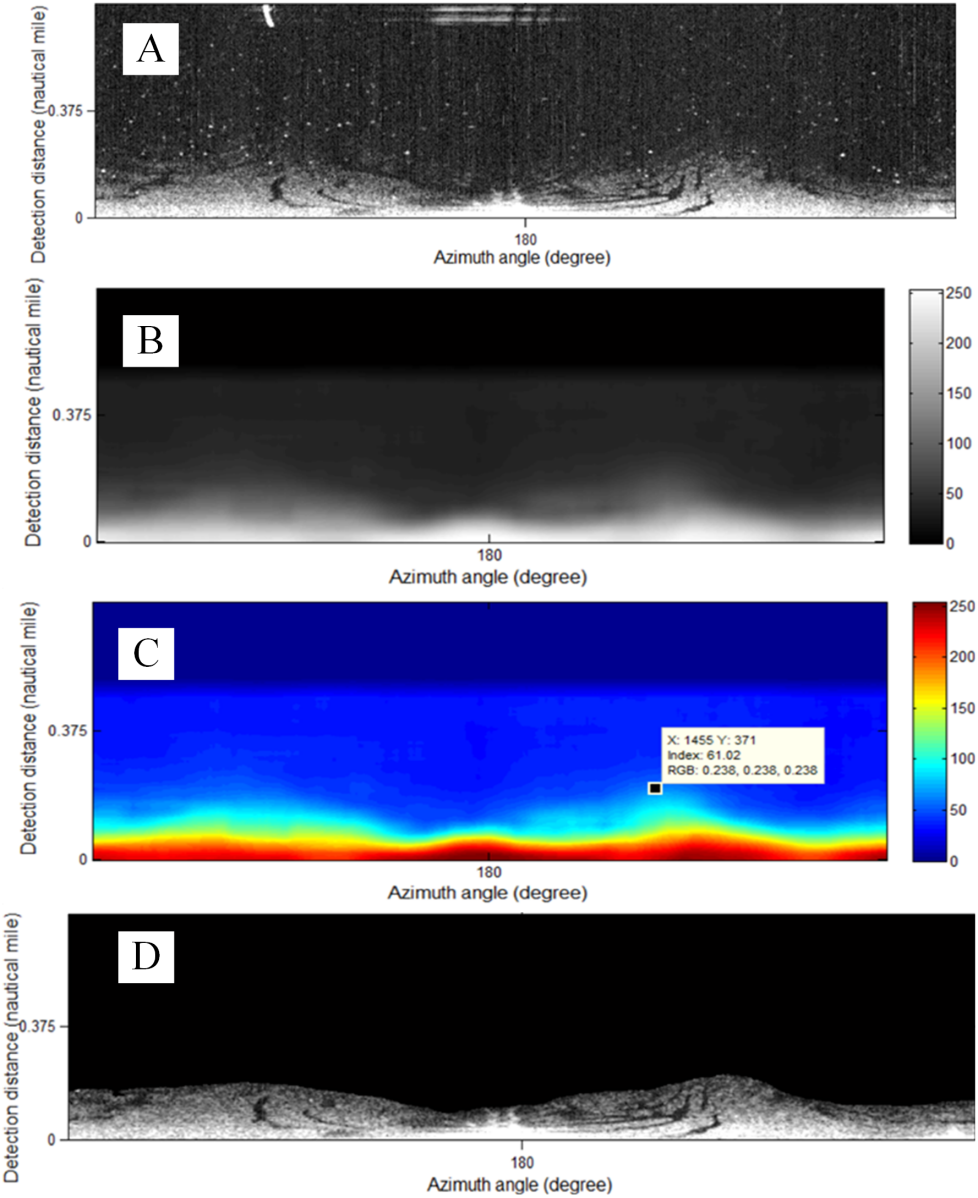
The effective wave range is set near the own ship. (A) Preprocessed image. (B) Gray distribution matrix. (C) Manual threshold selection in color scale image of (B). (D) Determination of effective wave range.

In the image after preprocessing, the information is deleted at a long distance (0.5 NM away in our experiment). Then, the image gray distribution matrix (Fig. 11B) is generated by convolution of a 20 ×80 window matrix. Next, the threshold (“61.02” in Fig. 11C) is selected in the color scale image of Fig. 11B to determine the effective wave range by visual interpretation and manual selection , as shown in Fig. 11D.

**Figure 12:**
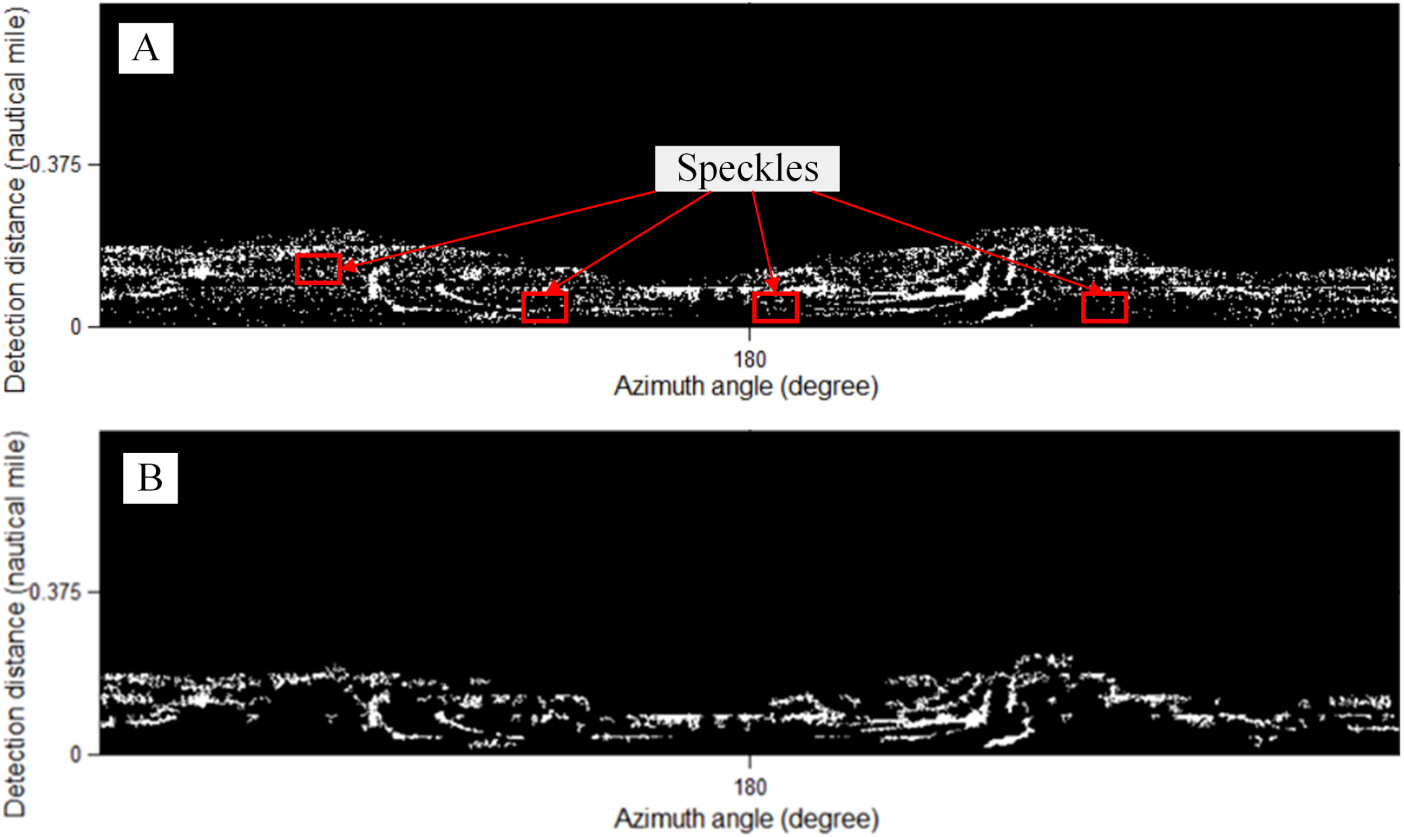
The local threshold segmentation. (A) Rough result of oil spill. (B) The speckles are removed.

The method of final segmentation is also more complicated than that of bright targets. Oil films can suppress the echo of sea waves and display them as relatively dark areas on waves in the images (Tong et al., 2019; Fingas & Brown, 2018). Therefore, local thresholds and active counter models are often used for segmentation (Liu et al., 2019). Figure 11D is segmented as Fig. 12 by the local threshold method (Xu et al., 2019) as:

(6) \begin{eqnarray*}T=m\times \left[ 1+k \left( \frac{v}{{R}^{2}} -1 \right) \right] ,\end{eqnarray*}

where *m* is the local mean, *k* is a user-defined parameter that takes negative values, *v* is the local standard deviation, and *R* is the dynamic range of the standard deviation. In our experiments, *k* = 0.25 and *R* = 128.

### Data fusion

**Figure 13:**
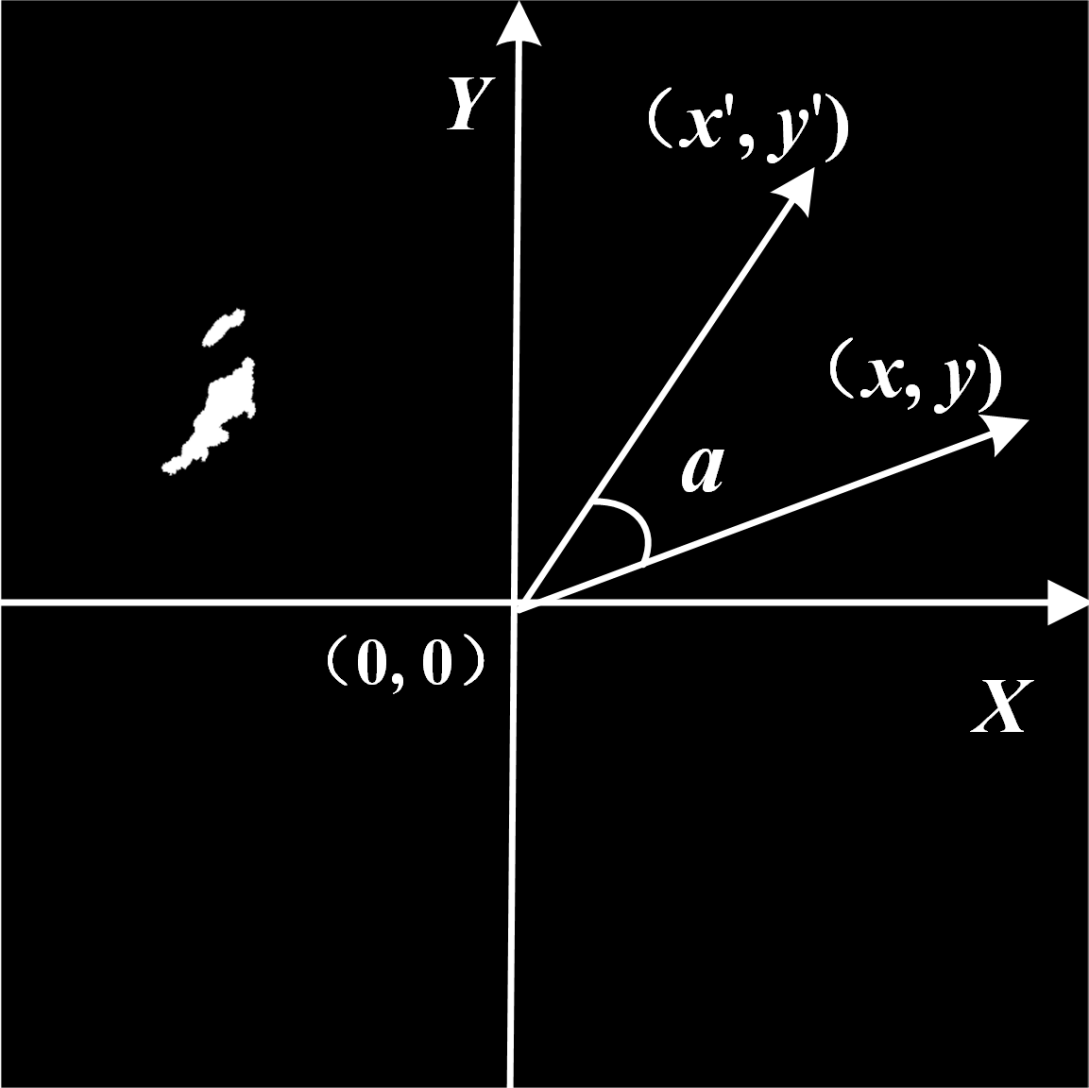
Image matrix coordinate system of Fig. 10B.

The image acquisition method of shipborne radars is head-up, and the ENC in our experiment is north up. To match the radar target with the ENC, the identification result needs to be rotated. An image matrix coordinate system was thus established to reduce the difficulty of image rotation, as shown in Fig. 13.

The image rotation formula is:

(7) \begin{eqnarray*} \left\{ \begin{array}{@{}l@{}} \displaystyle {x}^{{^{\prime}}}=x~cos \left( a \right) -y~sin \left( a \right) \\ \displaystyle {y}^{{^{\prime}}}=x~sin \left( a \right) +y~cos \left( a \right) \end{array} \right. \end{eqnarray*}

where *x*′ and *y*′ are the abscissa and ordinate after rotation, respectively; *x* and *y* are the abscissa and ordinate before rotation, respectively; and *a* is the angle of rotation.

**Figure 14:**
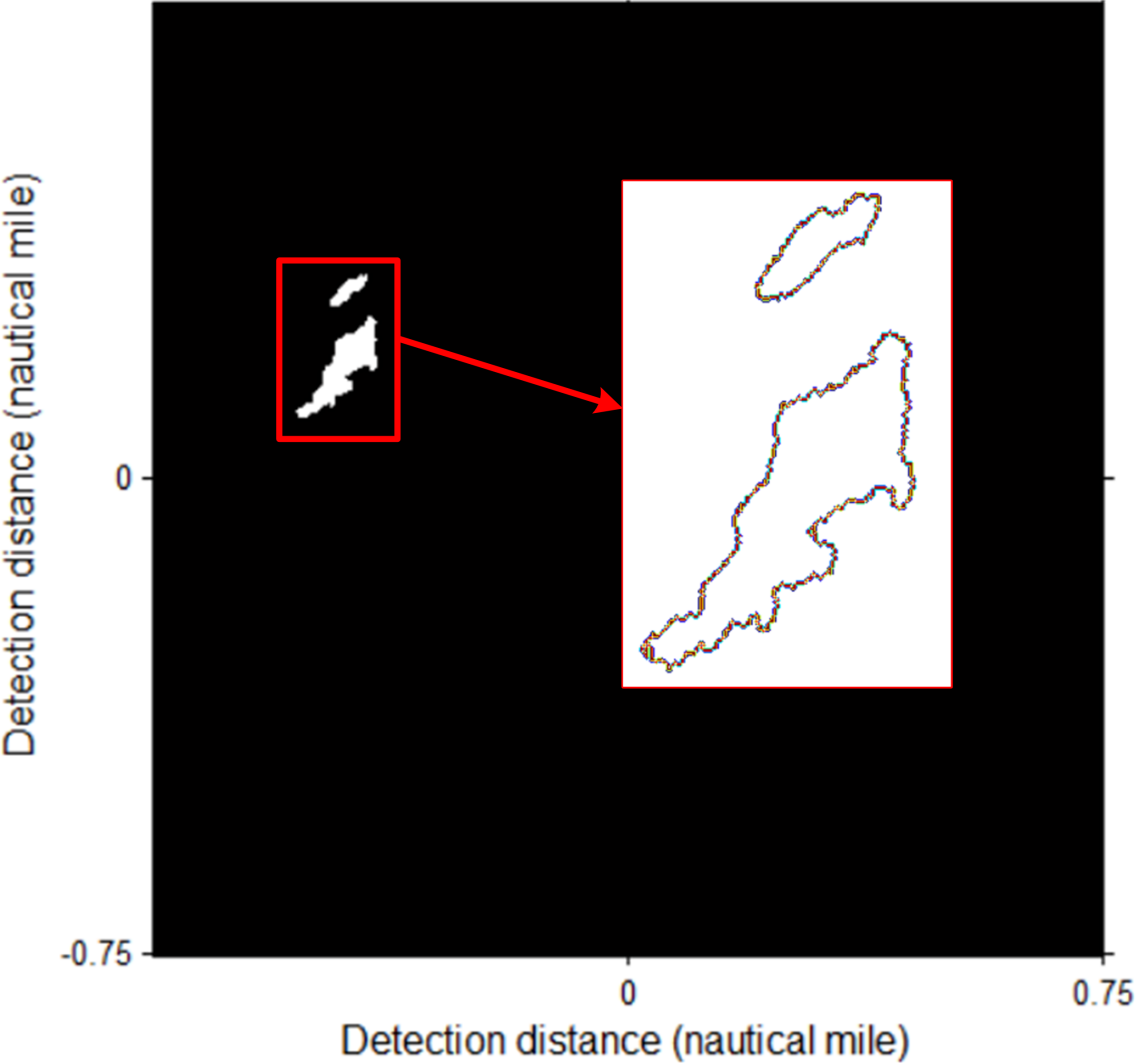
The counter extraction of coastline target. The image is rotated with heading direction (−19.5°).

The identification target is rotated with the angle according to the heading of the ship, and the counter is extracted as shown in Fig. 14.

The longitude and latitude coordinates of the ship can be obtained through GPS. In ArcGIS, the plane coordinate (*X*_*os*_, *Y*_*os*_) of the ship in meters can be obtained by projecting it from the WGS_1984 geographic coordinate system to the Beijing_1954 projection coordinate system. Based on the image matrix coordinates (*X*_*Pic*_, *Y*_*Pic*_), radar detection distance *D*, image radius *R*, and plane coordinates of the ship (*X*_*os*_, *Y*_*os*_), the plane coordinates (*X*_*BJ*_, *Y*_*BJ*_) of the target contour boundary point in the geodetic coordinate system of Beijing_1954 can be obtained as

(8) \begin{eqnarray*} \left\{ \begin{array}{@{}l@{}} \displaystyle {X}_{BJ}={X}_{os}+ \left( {X}_{Pic}/R \right) D\\ \displaystyle {Y}_{BJ}\,=\,{Y}_{os}+ \left( {Y}_{Pic}/R \right) D \end{array} \right. \end{eqnarray*}

Then through ArcGIS, target contour boundary points were converted back orderly to the geographic coordinates of the WGS_1984 coordinate system, as shown in Table 2.

**Figure 15:**
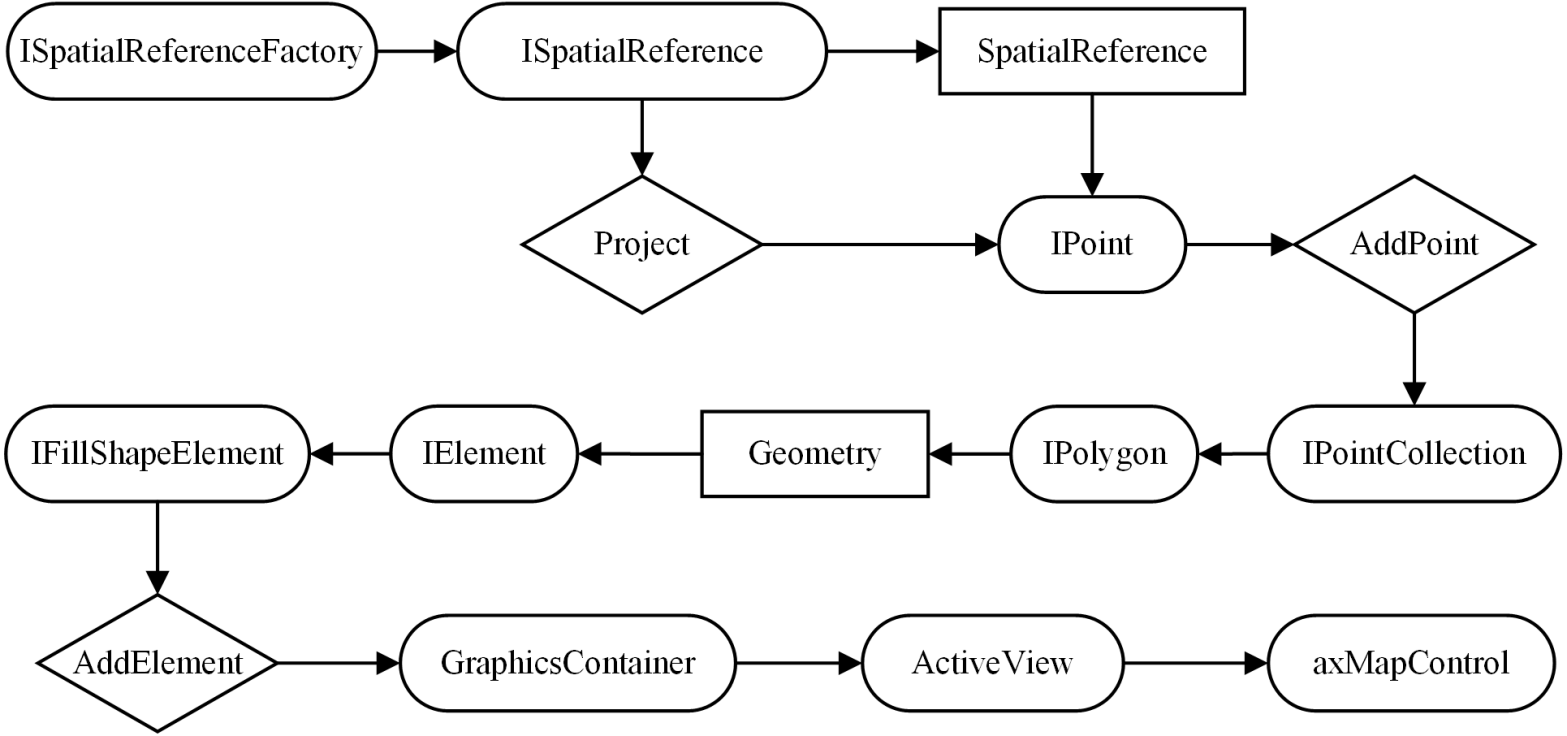
Object model diagram of space polygon generation in ArcGIS Objects.

After the geographical coordinates of the target boundary points were obtained, the target polygons were generated into an ENC based on ArcGIS Objects, as shown in Fig. 15. The detailed steps include the following: (a) According to the interfaces of ISpatialReferenceFactory and ISpatialReference, the properties of spatial reference (SpatialReference) and spatial projection (Project) of the target contour boundary point (IPoint) are set. (b) Using the AddPoint method, the boundary points are added to the point set (IPointCollection) to generate the target polygon. (c) The geometry attribute of the target polygon is assigned to the interface of the IElement. (d) Through the interface of IFillShapeElement, method of AddElement, class of GraphicsContainer and class of ActiveView, the target polygon is was added to the development control (axMapControl) of the ENC.

**Figure 16:**
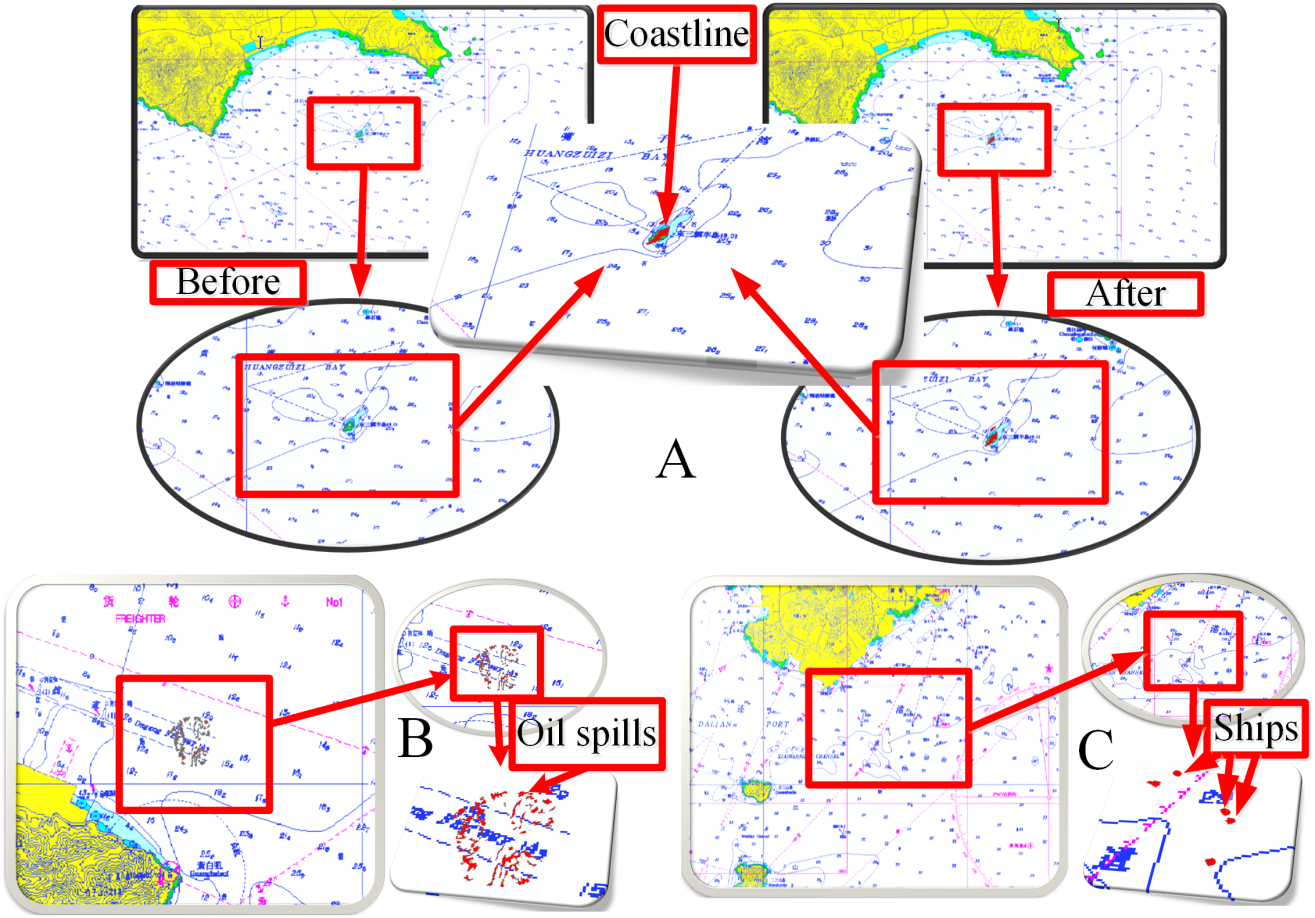
Information fusion. (A) Coastlines. (B) Oil spills. (C) Ships.

The coastline targets were fused into the ENC, as shown in Fig. 16A, which includes local information before and after fusion. The oil spill targets and ship targets were fused into the ENC, as shown Figs. 16B and 16C, respectively.

## Discussion

### Role of data fusion

The target data fusion between radars and the ENCs cannot only verify the hydrographic data but also assist the emergency command of marine disasters and accidents. Bright targets can be used to test the accuracy and timeliness of hydrographic data. For example, Fig. 16A can determine whether the island coastline data of the ENC are correct or whether the location of the lighthouse is correct. The coastline changes due to the influence of tides and storm surges. The island profile of low tide is shown in the ENC (green region in Fig. 16A). The island belongs to a strong reflector, so the radar can only detect its illuminated surface but not the blocked area. The radar detected that the coastline of the irradiating surface was beyond the island profile of the ENC. Thus, the ENC data must be updated in time.

**Table 2:** Geographical coordinates samples of coastline target boundary point. The GPS position of own ship is 122.0358°, 39.0319°.

Point ID	Longitude (°)	Latitude(°)
1	122.02595	39.03030
2	122.02598	39.03030
3	122.026015	39.030328
4	122.026015	39.030352
5	122.026014	39.030376
6	122.026046	39.030377

Dark targets can be used for marine disaster monitoring, such as the monitoring of oil spills, as shown in Fig. 16B. Remote sensors have become the main means of marine disaster monitoring technology. With respect to radar applications, airborne and spaceborne radars are maturely used for offshore disaster monitoring (Sankaran, 2019; Hammoud et al., 2019; Gallego et al., 2018). However, spaceborne remote sensing cannot conduct monitoring in real time. Airborne sensors cannot be used in severe weather conditions. The technology of shipborne radar marine disaster monitoring can overcome adverse weather conditions and remotely monitor real-time situations with high resolution. The data fusion of dark targets can provide technical support for the early warning and emergency disposal of marine disasters and accidents.

The ship target fusion of shipborne radars and ENC can provide a more effective guarantee for navigation safety. Our experimental data range is 0.75NM, as shown in Fig. 16C, which only provides a ship target fusion method for radars and the ENC. Real applications need to improve the detection range of radar images.

### Different segmentation methods for bright and dark targets

Ship and coastline targets have strong reflectivity, as highlighted in the images. Fixed thresholds can be used to segment them. However, oil film extraction is more difficult. A single threshold cannot identify discrete oil films in different positions. The local adaptive threshold is a better solution. In addition, because the database of the oil spill analysis is composed of waves, the monitoring range is close to the own ship, as shown in Fig. 11D. But, ships and coastlines are monitored for the inspection of hydrographic data. The monitoring area is located far away from the ship (Fig. 9).

### Comparison with other bright target segmentation methods for shipborne radar images

**Figure 17:**
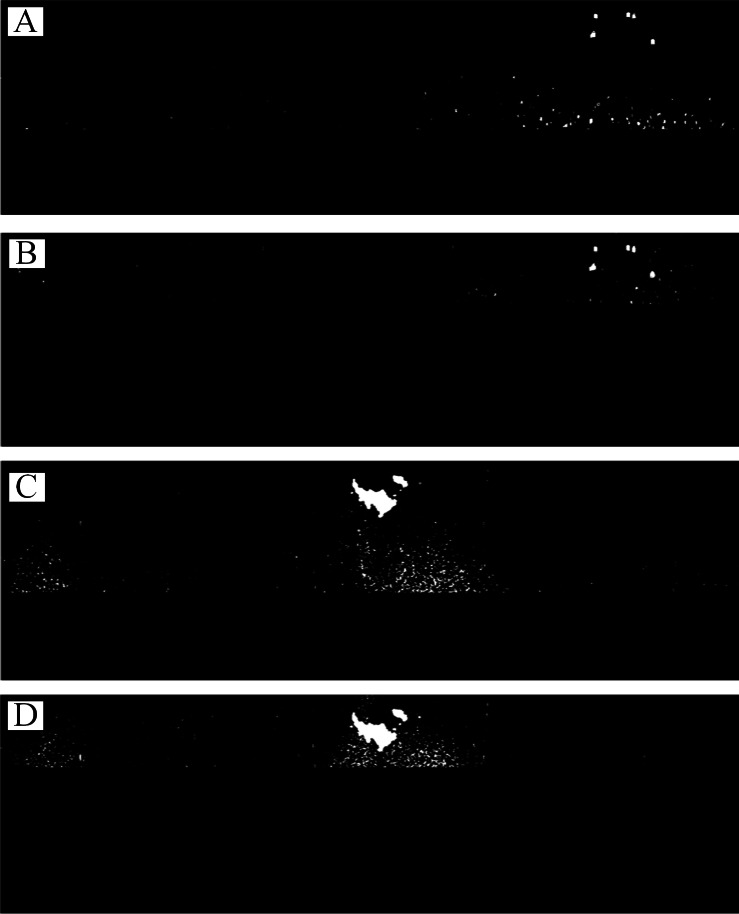
Rough bright targets extraction of Figs. 2B and 2C by Method 2. (A) and (B) are 0.3–0.75 nm and 0.5–0.75 nm monitoring area of Fig. 2B , respectively. (C) and (D) are 0.3–0.75 nm and 0.5–0.75 nm monitoring area of Fig. 2C, respectively.

The method of Wang et al. (2020) (hereafter referred as Method 2) and our method for extracting bright targets are compared here. Figure 2B and 2C are segmented using Method 2, as shown in Fig. 17.

Compared with Fig. 10A, only the preliminary extraction effect presented in Fig. 17B is similar. Too many false positive targets are present in Figs. 17C and 17D, implying that Method 2 is not suitable for large bright-target detection, such as islands and coastlines. Many false positive targets also exist, as shown Fig. 17A. For the complexity of the original image of the shipborne radar, Method 2 needs to set a smaller monitoring area to obtain good segmentation results for ships, as shown in Fig. 17B. Our method can identify ships and islands correctly, and set a large monitoring area, which has strong applicability in bright-target identification.

### Comparison with other dark target segmentation methods for shipborne radar images

**Figure 18:**
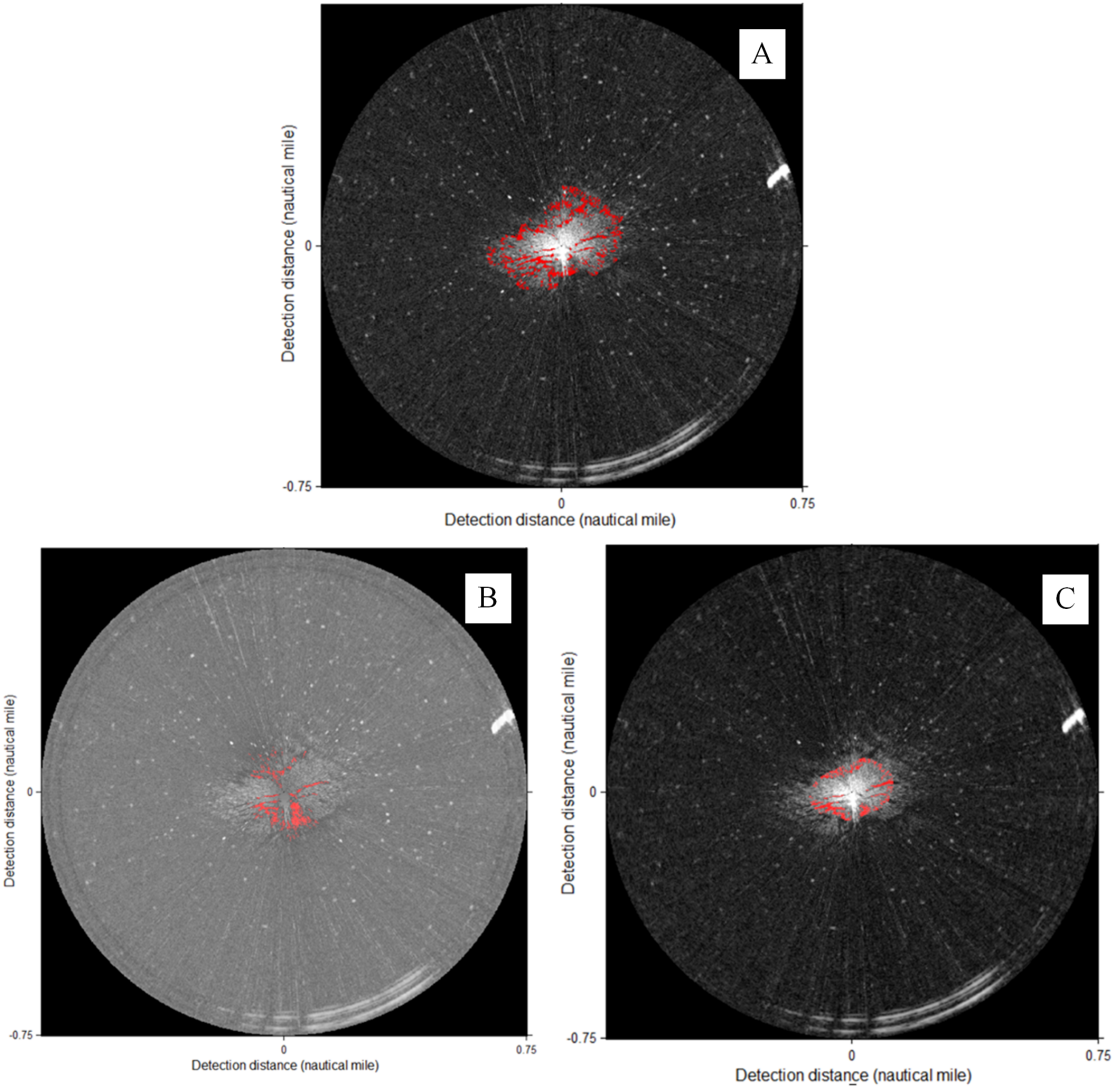
Comparison with other oil film segmentation methods of Fig. 2A. (A) our method. (B) Method 3 (*T*_*gray*_ = 100). (C) Method 4 (*T*_*gray*_ = 100, *T*_*area*_ = 30).

The methods of Zhu et al. (2015) (hereafter referred as Method 3), Xu et al. (2018) (hereafter referred as Method 4) and our method for extracting oil spills are compared with Fig. 2A in Fig. 18.

In Method 3, a global threshold method is proposed for oil film identification after gray adjustment, as shown in Fig. 18B. From the perspective of an expert interpretation, the recognition effect presented in Fig. 18B is not as good as that of our method. As the global threshold increases, Method 3 recognizes the false oil films in the area without wave information. In Method 4, a dual-threshold method is proposed to detect oil spills after choosing the wave range, as shown in Fig. 18C. Although a small wave range is selected, as shown in Fig. 18C, the segmentation effect is not good. As the wave range increases, the segmentation result becomes worse. Therefore, the major drawback of Method 4 is that it cannot select a further effective wave monitoring range. Therefore, the local adaptive threshold method is more suitable for the segmentation of large-range oil films and yields better results.

### Guarantee of navigation safety using the data fusion of an ENC and shipborne radar

The production of an ENC is time-consuming and has a slow update speed. Before sailing, the captain and pilot will create a general route using the information of the ENC. However, in the actual driving process, owing to the weak current situation of the ENC, the route will be made local adjustments in accurate information acquired by the radar.

The ENC can provide information on sunken ships, reefs, and shoals, among others. Shipborne radars can provide collision avoidance information for all other ships around the ship. After the data fusion of the ENC and shipborne radars, the dynamic information of a wider-range radar monitoring and the static information provided by the ENC can form a more efficient digital platform, which can improve the monitoring ability of pilot, and provide better guarantee for navigation safety.

### Lack of AIS data

At present, ENCs and shipborne radars have been fused with AIS system data. In radars and ENCs, the location and attribute information of a ship with AIS can be obtained (Jung, Kim & Song, 2007). However, the installation of AIS equipment is not required for small fishing boats or maintenance boats. Radars have an obvious marking effect on strong reflectors, which can highlight their reflecting surfaces on the screen. Therefore, the role of radar navigation and collision avoidance is essential. In addition, AIS targets use specific symbols in radars and ENCs, and there is no real shape. If the radar installation position of the ship is much higher than that of the target, then the target contour can be identified completely and integrated into the ENC. This characteristic makes up for the deficiency that AIS data cannot obtain the contour of the targets.

## Conclusions

Using the original shipborne radar images, this paper proposes methods for target extraction and fusion with ENCs for hydrographic data inspection and disaster monitoring. The experimental results show that proposed dual-threshold method can extract bright targets quickly and accurately. The local threshold method is more suitable for the complex image segmentation of dark targets. Finally, a data fusion method is proposed for shipborne radar images and ENC, which can be effectively used in hydrographic data inspection and marine disaster data monitoring.

It is noteworthy that the radar images used in this study have a relatively short range, which can meet the requirements of short-range hydrographic data inspection and auxiliary oil spill clean-up. In future work, we will increase the collection of radar images in a wider-range and improve the applicability of our method.

##  Supplemental Information

10.7717/peerj-cs.290/supp-1 Supplemental Information 1 Code

10.7717/peerj-cs.290/supp-2 Supplemental Information 2 Electronic chart

10.7717/peerj-cs.290/supp-3 Supplemental Information 3 Raw shipborne radar images

## Additional Information and Declarations

### Data Availability

The following information was supplied regarding data availability: The raw data are available in the Supplemental Files.
