# Supplementary figures and images for: Hydrographic data inspection and disaster monitoring using shipborne radar small range images with electronic navigation chart
Source: PeerJ Comput Sci. 2020 Sep 14;6:e290. doi: 10.7717/peerj-cs.290 (PMC7924651; doi:10.7717/peerj-cs.290)

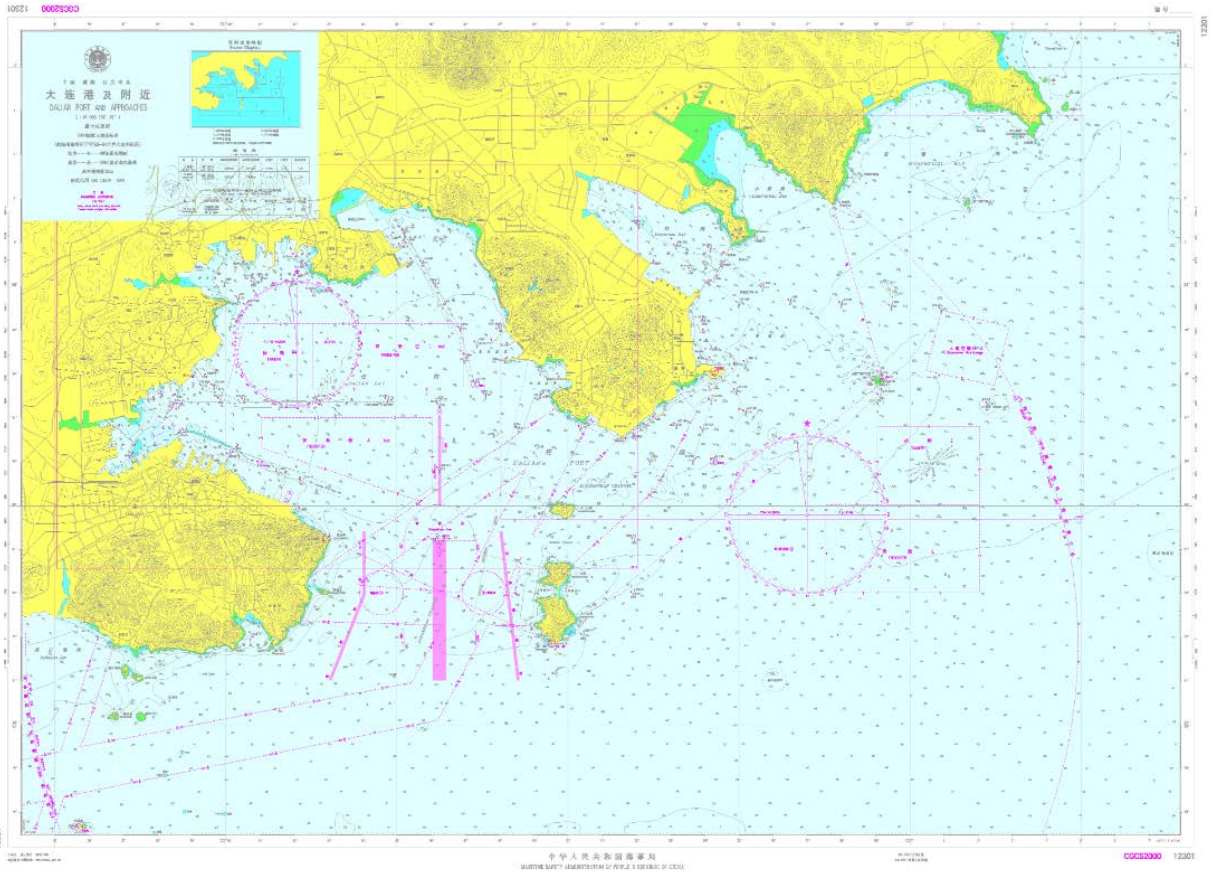

1  
2  
3

Supplement: Supplemental Information 2 [file peerj-cs-06-290-s002.pdf]

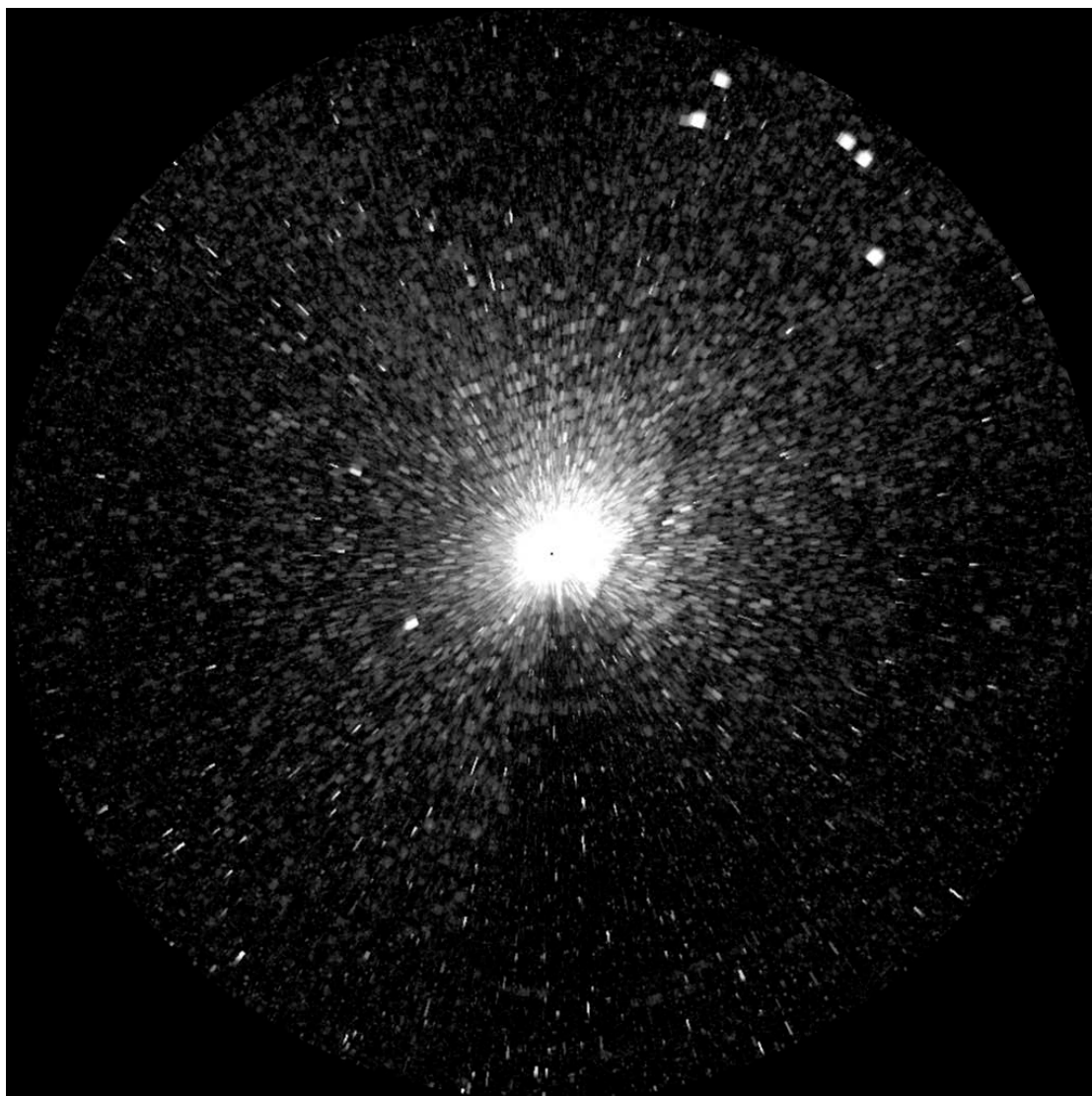

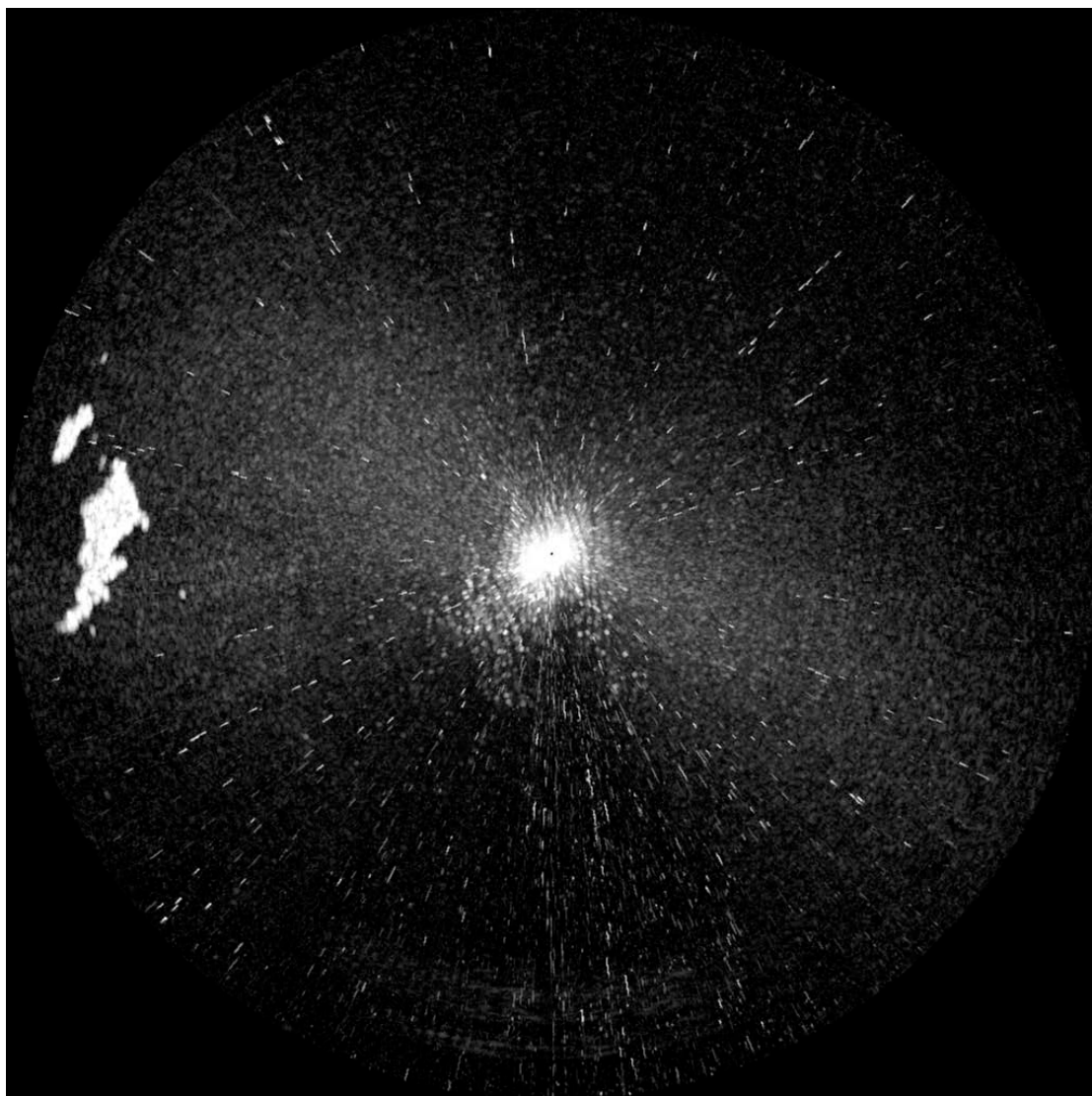

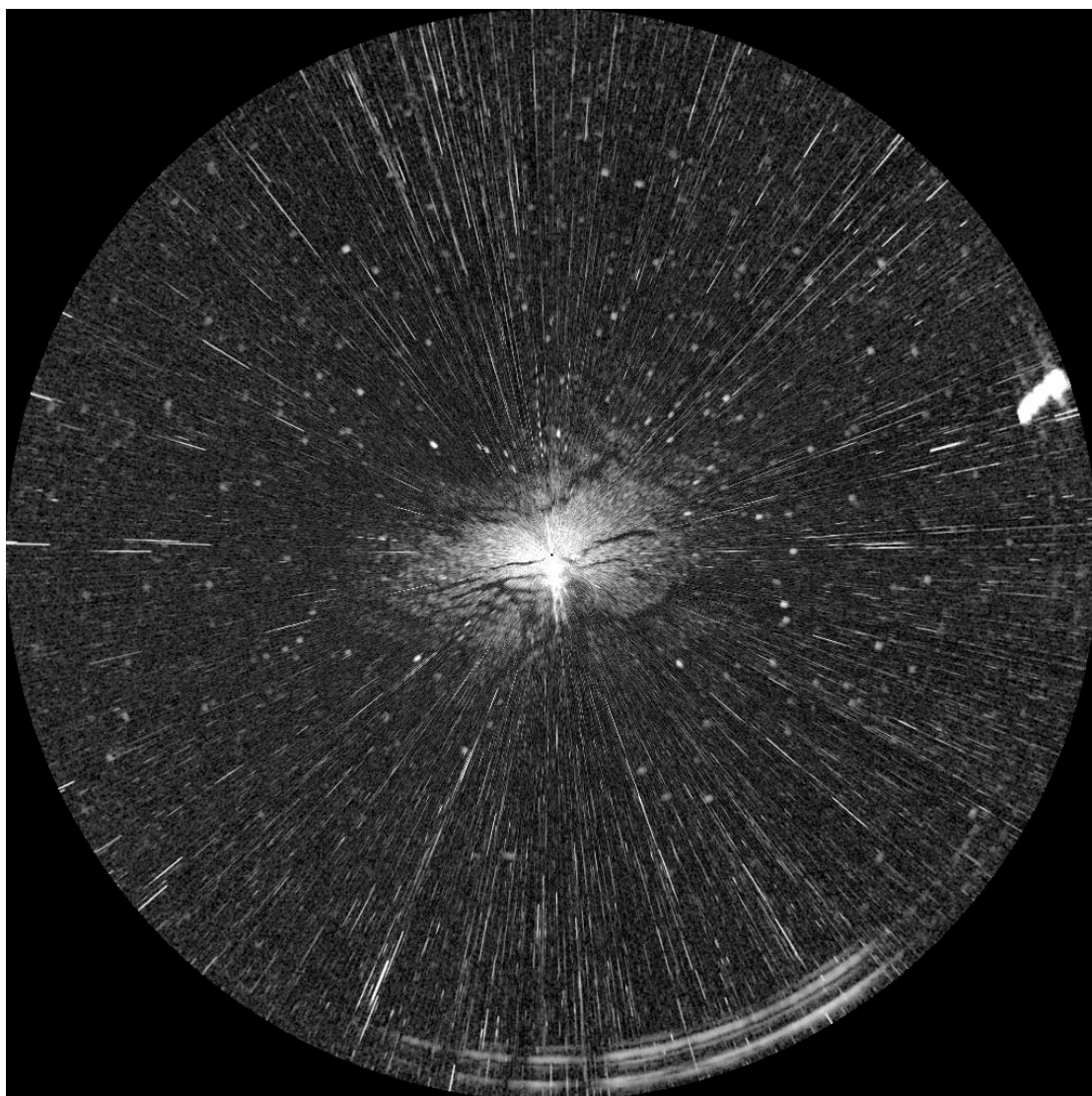

Supplement: Supplemental Information 3 [file peerj-cs-06-290-s003.pdf]
